# Social Vulnerability and Sickle Cell Disease Mortality in the US

**DOI:** 10.1001/jamanetworkopen.2024.40599

**Published:** 2024-09-30

**Authors:** Jia Yi Tan, Boon Jian San, Yong-Hao Yeo, Kok Hoe Chan, Hamid S. Shaaban, Daniel E. Ezekwudo, Modupe Idowu

**Affiliations:** 1 Department of Internal Medicine, New York Medical College at St Michael’s Medical Center, Newark, New Jersey; 2 Department of Internal Medicine, Jacobi Medical Center, Albert Einstein College of Medicine, Bronx, New York; 3 Department of Internal Medicine and Pediatrics, Corewell Health William Beaumont University Hospital, Royal Oak, Michigan; 4 Division of Hematology and Oncology, Department of Internal Medicine, McGovern Medical School at the University of Texas Health Science Center at Houston (UTHealth Houston); 5 Division of Hematology and Oncology, Department of Internal Medicine, New York Medical College at St Michael’s Medical Center, Newark, New Jersey; 6 Department of Hematology and Oncology, Oakland University William Beaumont School of Medicine, Royal Oak, Michigan; 7 Division of Hematology, Department of Internal Medicine, McGovern Medical School at the University of Texas Health Science Center at Houston (UTHealth Houston)

## Abstract

**Importance:**

Social determinants of health (SDOH) influence health outcomes, including those of sickle cell disease (SCD), despite advancements in treatments like disease-modifying therapies.

**Objective:**

To investigate the association of SDOH with SCD mortality rates from 2016 to 2020.

**Design, Setting, and Participants:**

This cross-sectional study combined county-level data from the Centers for Disease Control and Prevention and Agency for Toxic Substances and Disease Registry Social Vulnerability Index (SVI) with SCD mortality data from the Centers for Disease Control and Prevention Wide-Ranging Online Data for Epidemiologic Research database from January 1, 2016, to December 31, 2020. US counties were divided into 4 quartile (Q) models according to their SVI scores. Deaths from SCD in the US among patients of all ages were included. Data analysis occurred from March to April 2024.

**Exposure:**

SVI score.

**Main Outcomes and Measures:**

Age-adjusted mortality rates (AAMRs) per 1 000 000 individuals were measured. Rate ratios (RRs) were obtained by comparing county-specific AAMRs of SVI-Q4 with SVI-Q1.

**Results:**

From 2016 to 2020, among a total population of 1 633 737 771 individuals, there were 2635 deaths from SCD (1289 male [49.1%] and 1336 female [50.9%]). There were 1480 deaths in Q4, 687 deaths in Q3, 344 deaths in Q2, and 114 deaths in Q1. Higher SVI was associated with 2.11 excess deaths per 1 000 000 individuals (RR, 4.90; 95% CI, 4.81-5.00). Similar trends were seen for both males (RR, 4.56; 95% CI, 4.44-4.69) and females (RR, 5.85; 95% CI, 5.68-6.03). Middle-aged patients with SCD had the highest mortality rate in Q4, with 3.45 excess deaths per 1 000 000 individuals (RR, 4.97; 95% CI, 4.85-5.09). Higher SVI was associated with 2.29 excess deaths per 1 000 000 individuals in African American individuals with SCD (RR, 1.24; 95% CI, 1.22-1.27]). In White individuals with SCD, higher SVI was associated with 0.12 excess deaths per 1 000 000 individuals (RR not available due to unreliable data in Q1). When stratifying by census region, the highest level of SCD-related mortality was in the Northeast, with higher SVI associated with 3.16 excess deaths per 1 000 000 individuals (RR, 8.02; 95% CI, 7.66-8.40).

**Conclusions:**

In this cross-sectional study of the association of SVI with SCD mortality rates, higher SVI was associated with higher SCD mortality across US counties. These findings underscore the importance of addressing social determinants of health to improve mortality outcomes among patients with SCD.

## Key Points

**Question:**

What is the association of social determinants of health with sickle cell disease mortality?

**Findings:**

In this cross-sectional study that included 2625 adults with sickle cell disease, patients with the highest social vulnerability index score had a 4.9 times higher mortality risk compared with patients with the lowest social vulnerability index score.

**Meaning:**

These findings suggest that addressing the social determinants of health among patients with sickle cell disease may improve the mortality rate.

## Teaser

This cross-sectional study analyzes the association of social determinants of health with sickle cell disease–related mortality among individuals in the US.

## Introduction

Sickle cell disease (SCD) is one of the hereditary hemoglobinopathies arising from a missense variant within the hemoglobin subunit-β gene, which encodes the β-globin subunit of hemoglobin.^1^ This variant results in the formation of sickled-shaped erythrocytes, which are prone to hemolysis.^2^

It was estimated that approximately 100 000 individuals in the US have SCD.^3^ Approximately 1 of every 365 African American individuals are born with SCD in the US^4^ The average annual death rate due to SCD among children younger than 5 years declined from 2.05 per 100 000 individuals from 1979 to 1989 to 0.47 per 100 000 individuals from 2015 to 2017 in the US. Conversely, for adults aged 60 years and older, the rate increased from 1.20 per 100 000 individuals to 1.99 per 100 000 individuals.^5^

As early as 1998, the US Food and Drug Administration approved hydroxyurea as a disease-modifying agent for SCD.^6^ A 2003 study demonstrated the significant benefit of starting hydroxyurea in patients with SCD, reducing their mortality by 40%.^7^ Despite the proven benefits of hydroxyurea, there remain barriers regarding its use,^8^ such as clinicians’ hesitancy in prescribing hydroxyurea;^9^ patients’ lack of adherence due to forgetfulness, negative perception of hydroxyurea, or reluctance to take any medication;^10^ and disadvantaged socioeconomic status.^11^

Since 2017, newer therapy agents, including L-glutamine, crizanlizumab, and voxelotor, have also emerged as additional disease-modifying therapies.^12^ Despite the advancements in the treatment of SCD, the accessibility of the novel agents remains questionable.^13,14^ It is crucial to note that the advances in treatment do not necessarily translate into improved population-level mortality rates due to hindering factors, notably social determinants of health. Deenadayalan and colleagues^15^ found lower socioeconomic status was associated with lower reductions in mortality rates among patients with SCD. Another study^16^ reported negative experiences among patients with SCD such as “worrying about cost” and “previous bad experiences with the healthcare system.”

Given the uncertain influence of social determinants of health on SCD outcomes, our study aimed to investigate the association of social determinants of health with SCD mortality rates using the social vulnerability index (SVI) dataset. SVI is a composite measure that was used to identify populations at higher risk of mortality from different chronic illnesses including diabetes-related cardiovascular disease,^17^ premature cardiovascular disease,^18^ and chronic respiratory diseases such as asthma and chronic obstructive lung disease.^19^ With the rapid advancements in disease-modifying therapies, understanding the associations of mortality rates with SVI scores is crucial to determine whether these advancements translate to mortality benefits across all populations in the US, taking their SVI status into account. We sought to provide insights that could inform strategies for improving outcomes and addressing health care delivery disparities for patients with SCD.

## Methods

### Data Source

This cross-sectional study did not require institutional review board approval or patient informed consent because it used publicly available deidentified data in accordance with 45 CFR § 46. The reporting of the study followed the Strengthening the Reporting of Observational Studies in Epidemiology (STROBE) reporting guideline. We analyzed death certificate data from the US Centers for Disease Control and Prevention Wide-Ranging Online Data for Epidemiologic Research (CDC WONDER) from January 1, 2016, to December 31, 2020, to determine the longitudinal trends of SCD mortality among the US population of all ages. CDC WONDER is a publicly available online database containing public health data, including mortality data, since 1999. Patients with SCD-related mortality were identified using the *International Statistical Classification of Diseases and Related Health Problems, Tenth Revision (ICD-10)* codes. SCD (*ICD-10* code D57) was identified as the underlying cause of death. The World Health Organization defines the underlying cause of death as the disease or injury that initiates a sequence of events that leads directly to death.^20^

We obtained the 2018 SVI dataset from the CDC Agency for Toxic Substances and Disease Registry Geospatial Research, Analysis, and Services Program. This index indicates the social vulnerability of US census tracts based on 15 social factors using data from the American Community Survey from January 1, 2016, to December 31, 2020, as shown in eFigure 1 in Supplement 1. There are 4 social factor categories: socioeconomic status, household composition and disability, minority status and primary language, and housing type and transportation. The SVI ranges from 0 to 1, with 0 representing the lowest vulnerability and 1 representing the highest vulnerability. US counties were divided into 4 quartile (Q) models for SVI scores (SVI-Q1, 0.00-0.25; SVI-Q2, 0.26-0.50; SVI-Q3, 0.51-0.75; SVI-Q4, 0.76-1.00) using the overall SVI (eFigure 2 in Supplement 1). This study methodology has been validated in similar research topics.^17,18,19^

### Statistical Analysis

We analyzed the age-adjusted mortality rates (AAMRs) per 1 000 000 individuals, standardizing to the 2000 US census population. We plotted the AAMR per 1 000 000 individuals to determine the trends from 2016 to 2020. We also compared the AAMRs in different sexes, ages, ethnicity, races, geographic regions, and urbanization levels. Ethnicity and race were assessed to study how the SVI is associated with SCD mortality rates within specific ethnic and racial groups and to ensure the comprehensiveness of data analyses. The study utilized ethnicity and race data recorded on death certificates, as captured in CDC WONDER. Ethnicity categories included Hispanic and non-Hispanic, while race categories included African American and White populations. Urbanization level was categorized into metropolitan and nonmetropolitan regions. Metropolitan regions included large central metropolitan regions, large fringe metropolitan regions, medium metropolitan regions, and small metropolitan regions. Nonmetropolitan regions included micropolitan regions and noncore nonmetropolitan regions.^21,22^

We measured rate ratios (RRs) by comparing county-specific AAMRs with the highest vulnerability (SVI-Q4) to those with the lowest vulnerability (SVI-Q1) for the overall population. This analysis was done using Poisson univariable regression. A risk ratio with a 95% CI that does not cross 1 was considered statistically significant. We completed data analysis and visualization using Stata statistical software version 17.0 (StataCorp) and Python version 3.9.6 (Python Software Foundation). Data analysis was conducted from March to April 2024.

## Results

From 2016 to 2020, among a total population of 1 633 737 771 individuals, there were 2625 deaths related to SCD (1289 male [49.1%] and 1336 female [50.9%]). Most of the SCD-related deaths occurred among patients aged 25 to 54 years (1705 patients [65.0%]), followed by patients aged 55 years or older (606 patients [23.0%]), and patients aged younger than 25 years (314 patients [12.0%]). Of all SCD-related deaths, 2505 (95.4%) occurred among non-Hispanic patients and 2480 (94.5%) occurred among African American patients. The absolute number of deaths for Hispanic patients and other racial groups was unavailable due to confidentiality protocols, which required censoring because of the low case number. Most of the SCD-related deaths occurred in metropolitan regions (2360 deaths [89.9%]), and the rest occurred in nonmetropolitan regions (265 deaths [10.1%]). The highest number of SCD-related deaths was in the South region (1523 deaths [58.0%]), followed by the Midwest (441 deaths [16.8%]), the Northeast (435 deaths [16.6%]), and the West (225 deaths [8.6%]).

There were 1480 deaths in Q4, 687 deaths in Q3, 344 deaths in Q2, and 114 deaths in Q1. Higher SVI was associated with 1366 excess deaths in the US. SCD-related mortality decreased from 2016 (AAMR, 1.67 per 1 000 000 individuals; 95% CI, 1.52-1.81 per 1 000 000 individuals) to 2018 (AAMR, 1.60 per 1 000 000 individuals; 95% CI, 1.46-1.74 per 1 000 000 individuals), and then increased in 2020 (AAMR, 1.67 per 1 000 000 individuals; 95% CI, 1.53-1.82 per 1 000 000 individuals). Overall, the highest level of SCD-related mortality was reported in Q4 (AAMR, 2.65 per 1 000 000 individuals; 95% CI, 2.52-2.79 per 1 000 000 individuals), whereas the lowest was reported in Q1 (AAMR, 0.54 per 1 000 000 individuals; 95% CI, 0.44-0.64 per 1 000 000 individuals), with higher SVI was associated with 2.11 excess deaths per 1 000 000 individuals (RR, 4.90; 95% CI, 4.81-5.00) (Table).

**Table.** Baseline Characteristics of Sickle Cell Disease Deaths, AAMR per 1 000 000 by SVI Across US Counties, and Their RRs, 2016 to 2020^a^

Population	SVI-Q1		SVI-Q2		SVI-Q2 vs SVI-Q1, RR (95% CI)	SVI-Q3		SVI-Q3 vs SVI-Q1, RR (95% CI)	SVI-Q4		SVI-Q4 vs SVI-Q1, RR, 95% CI)
	Deaths, No. (%)	AAMR (95% CI)	Deaths, No. (%)	AAMR (95% CI)		Deaths, No. (%)	AAMR (95% CI)		Deaths, No. (%)	AAMR (95% CI)	
Total deaths, No.	114	0.54 (0.44-0.64)	344	0.87 (0.78-0.97)	1.61 (1.30-2.00)	687	1.52 (1.40-1.64)	2.81 (2.30-3.45)	1480	2.65 (2.52-2.79)	4.90 (4.81-5.00)
Sex											
Male	60 (52.6)	0.57 (0.44-0.74)	186 (54.1)	0.97 (0.82-1.11)	1.70 (1.26-2.30)	344 (50.1)	1.57 (1.40-1.74)	2.75 (2.07-3.66)	699 (47.2)	2.60 (2.40-2.79)	4.56 (4.44-4.69)
Female	54 (47.4)	0.47 (0.35-0.63)	158 (45.9)	0.79 (0.66-0.91)	1.68 (1.21-2.33)	343 (49.9)	1.48 (1.32-1.64)	3.15 (2.32-4.27)	781 (52.8)	2.75 (2.56-2.95)	5.85 (5.68-6.03)
Age groups, y											
<25	14 (12.3)	Unreliable	51 (14.8)	0.40 (0.30-0.53)	Baseline	70 (10.2)	0.45 (0.35-0.56)	1.13 (0.77-1.63)	179 (12.1)	0.93 (0.79-1.06)	2.07 (2.01-2.13)^b^
24-54	70 (61.4)	0.87 (0.68-1.10)	203 (59.0)	1.33 (1.15-1.52)	1.52 (1.17-2.00)	469 (68.3)	2.63 (2.39-2.87)	3.02 (2.36-3.88)	963 (65.1)	4.32 (4.04-4.59)	4.97 (4.85-5.09)
≥55	30 (26.3)	0.44 (0.29-0.63)	90 (26.2)	0.73 (0.59-0.90)	1.66 (1.10-2.51)	148 (21.5)	1.04 (0.87-1.21)	2.36 (1.60-3.50)	338 (22.8)	2.14 (1.91-2.37)	4.86 (4.69-5.05)
Ethnicity											
Hispanic	<10 (<10.0%)	Unreliable	10 (2.9)	Unreliable	NA	648 (94.3)	0.44 (0.29-0.64)	NA	1396 (94.3)	0.43 (0.34-0.54)	NA
Non-Hispanic	111 (97.4)	0.56 (0.45-0.67)	334 (97.1)	0.93 (0.83-1.03)	1.66 (1.33-2.07)	657 (95.6)	1.76 (1.62-1.90)	3.14 (2.56-3.86)	1403 (94.8)	3.67 (3.47-3.86)	6.55 (6.20-6.93)
Race											
African American	109 (95.6)	9.48 (7.67-11.29)	327 (95.1)	8.71 (7.75-9.67)	0.92 (0.74-1.15)	648 (94.3)	11.39 (10.50-12.28)	1.20 (0.98-1.48)	1396 (94.3)	11.77 (11.14-12.40)	1.24 (1.22-1.27)
White	<10 (<10.0%)	Unreliable	16 (4.7)	Unreliable	NA	35 (5.1)	0.09 (0.06-0.13)	NA	77 (5.2)	0.21 (0.16-0.26)	NA
Urbanization											
Metropolitan	101 (88.6)	0.59 (0.47-0.71)	330 (95.9)	0.95 (0.84-1.05)	1.61 (1.28-2.02)	643 (93.6)	1.63 (1.50-1.76)	2.76 (2.23-3.42)	1286 (86.9)	2.61 (2.46-2.75)	4.42 (4.33-4.52)
Nonmetropolitan	13 (11.4)	Unreliable	14 (4.1)	Unreliable	NA	44 (6.4)	0.77 (0.56-1.05)	NA	194 (13.1)	2.97 (2.54-3.40)	NA
US census region											
Northeast	21 (18.4)	0.45 (0.27-0.71)	63 (18.3)	0.73 (0.56-0.94)	1.62 (0.98-2.69)	109 (15.9)	1.44 (1.16-1.72)	3.20 (1.98-5.17)	242 (16.4)	3.61 (3.15-4.07)	8.02 (7.66-8.40)
Midwest	56 (49.1)	0.52 (0.39-0.67)	66 (19.2)	0.73 (0.57-0.94)	1.40 (0.98-2.02)	152 (22.1)	1.92 (1.61-2.24)	3.69 (2.70-5.04)	167 (11.3)	3.04 (2.57-3.51)	5.85 (5.67-6.03)
South	37 (32.5)	1.02 (0.71-1.41)	188 (54.7)	1.47 (1.25-1.68)	1.44 (1.00-2.07)	352 (51.2)	2.14 (1.91-2.37)	2.10 (1.48-2.97)	946 (63.9)	3.39 (3.17-3.61)	3.32 (3.21-3.44)
West	0	Unreliable	27 (7.9)	0.31 (0.20-0.45)	Baseline	74 (10.8)	0.57 (0.45-0.72)	1.84 (0.42-8.07)	125 (8.5)	0.81 (0.67-0.96)	2.61 (2.26-3.03)^a^

Abbreviations: AAMR, age-adjusted mortality rates; NA, not applicable; SVI, social vulnerability index; Q, quartile; RR, rate ratio.

^a^ US counties were divided into 4 quartiles for SVI scores (SVI-Q1, 0.00-0.25; SVI-Q2, 0.26-0.50; SVI-Q3, 0.51-0.75; SVI-Q4, 0.76-1.00).

^b^ RR calculated using SVI- Q2 instead of SVI-Q1 as the data was marked as unreliable in the database.

### Sex and Age Groups

SCD-related mortality was higher in males than females, although the difference was not significant (AAMR, 1.64 per 1 000 000 individuals; 95% CI, 1.55-1.73 per 1 000 000 individuals vs AAMR, 1.62 per 1 000 000 individuals; 95% CI, 1.53-1.71 per 1 000 000 individuals) (eTable in Supplement 1). Both sexes exhibited higher mortality rates with higher SVI. In males, higher SVI was associated with 2.03 excess deaths per 1 000 000 individuals (RR, 4.56; 95% CI, 4.44-4.69). In females, higher SVI was associated with 2.28 excess deaths per 1 000 000 individuals (RR, 5.85; 95% CI, 5.68-6.03) (Table).

Among 3 different age groups, higher SVI was associated with higher mortality rates. Higher SVI was associated with 0.53 excess deaths per 1 000 000 individuals in individuals younger than 25 years (RR, 2.07; 95% CI, 2.01-2.13), 3.45 excess deaths per 1 000 000 individuals in individuals 25 to 54 years (RR, 4.97; 95% CI, 4.85-5.09), and 1.70 excess deaths per 1 000 000 individuals in individuals 55 years or older (RR, 4.86; 95% CI, 4.69-5.05) (Table).

### Ethnicity and Race

Non-Hispanic patients also exhibited higher SCD-related mortality (AAMR, 1.93 per 1 000 000 individuals; 95% CI, 1.85-2.00 per 1 000 000 individuals) compared with Hispanic patients (AAMR, 0.38 per 1 000 000 individuals; 95% CI, 0.31-0.45 per 1 000 000 individuals). SCD-related mortality increased in non-Hispanic patients with an increasing SVI score. Non-Hispanic populations had a higher level of SCD-related mortality in Q4 (AAMR, 3.67 per 1 000 000 individuals; 95% CI, 3.47-3.86 per 1 000 000 individuals) compared with Q1 (AAMR, 0.56 per 1 000 000 individuals; 95% CI, 0.45-0.67 per 1 000 000 individuals), with a higher SVI associated with 3.11 excess deaths per 1 000 000 individuals (RR, 6.55; 95% CI, 6.20-6.93) (Table).

Among different racial groups, higher SCD-related mortality was observed among African American patients (AAMR, 11.91 per 1 000 000 individuals; 95% CI, 11.69-12.14 per 1 000 000 individuals) compared with White patients (AAMR, 0.09 per 1 000 000 individuals; 95% CI, 0.08-0.10 per 1 000 000 individuals). Both African American and White individuals demonstrated greater mortality rates in counties with higher SVI. Higher SVI was associated with 2.29 excess deaths per 1 000 000 individuals in African American individuals (RR, 1.24; 95% CI, 1.22-1.27]) and 0.12 excess deaths per 1 000 000 individuals in White individuals (RR not available due to unreliable data in Q1 and Q2).

### Geographic Region

Metropolitan regions had higher SCD-related mortality than nonmetropolitan regions (AAMR, 1.69 per 1 000 000 individuals; 95% CI, 1.62-1.76 per 1 000 000 individuals vs 1.25 per 1 000 000 individuals; 95% CI, 1.10-1.41 per 1 000 000 individuals). High SVI was associated with increased mortality in metropolitan and nonmetropolitan regions. In metropolitan regions, higher SVI was associated with 2.02 excess deaths per 1 000 000 individuals. Likewise, nonmetropolitan regions had higher SCD-related mortality in Q4 (AAMR, 2.97 per 1 000 000 individuals; 95% CI, 2.54-3.40 per 1 000 000 individuals) compared with Q3 (AAMR, 0.77 per 1 000 000 individuals; 95% CI, 0.56-1.05 per 1 000 000 individuals) with a higher SVI associated with 2.20 excess deaths per 1 000 000 individuals (RR not available due to unreliable data in Q1 and Q2).

The highest level of SCD-related mortality was observed in the South region (AAMR, 2.51 per 1 000 000 individuals; 95% CI, 2.38-2.64 per 1 000 000 individuals), whereas the lowest level was shown in the West region (AAMR, 0.59 per 1 000 000 individuals; 95% CI, 1.58-1.71 per 1 000 000 individuals). When stratified by different census regions, higher SVI was associated with 3.16 excess deaths per 1 000 000 individuals in the Northeast region (RR, 8.02; 95% CI, 7.66-8.40]), 2.52 excess deaths per 1 000 000 individuals in the Midwest region (RR, 5.85; 95% CI, 5.67-6.03), 2.37 excess deaths per 1 000 000 individuals in the South region (RR, 3.32; 95% CI, 3.21-3.44]), and 0.50 excess deaths per 1 000 000 individuals in the West region (RR, 2.61; 95% CI, 2.26-3.03).

## Discussion

The results of this cross-sectional study were based on the 5-year CDC WONDER database analyses from 2016 to 2020. Our analyses showed greater SCD mortality in counties with higher SVI, both collectively and across various demographic subsets. SCD mortality in Q4 was higher in males than females, although the difference was insignificant. Among different age groups, individuals with SCD between 25 and 44 years exhibited the highest mortality in Q4. Both metropolitan and nonmetropolitan regions reported a higher AAMR in counties with higher SVI.

Our study demonstrated that greater SVI is associated with higher mortality rates from SCD. Socioeconomic status plays a vital role in health outcomes among patients with SCD. As one of the most common inherited blood disorders in the US, it has been found that individuals with SCD are less likely to receive comprehensive care, especially those residing in low-income and rural areas.^23^ The SVI incorporates unemployment as one of the factors. A study by Williams and colleagues^24^ found that unemployment was associated with more emergency department (ED) visits and hospital admissions among patients with SCD. A study by Maitra et al^25^ indicated that the mortality rate increased with the number of ED visits for each additional pain episode among patients with SCD. This finding can be explained by the slower decline in perceived health and physical functioning among those with full-time employment compared to those without.^24^

Additionally, poor household composition (ie, households consisting of families with members aged 65 years or older or 17 years or younger, civilians with disabilities, or single-parent families) is also associated with higher mortality among patients with SCD. This finding is supported by a study^24^ that demonstrated unstable home conditions were associated with a higher number of ED visits and hospital admissions. Unstable home conditions were associated with higher psychological stress, depression, and anxiety.^26^ Onyeaka et al^27^ found comorbid depression was significantly associated with longer length of stay and increased severity of illness among patients with SCD. This finding can be explained by the higher burden of comorbid diseases such as cardiovascular disease,^28^ diabetes,^29^ and migraine^30^ among patients with depression. Hence, we propose that improving home conditions may help mitigate the impact of SVI on SCD mortality.

Our study indicated the highest number of deaths in the South region, which may be related to higher SCD prevalence in this area as shown in a study by Kanter and colleagues.^31^ The limited accessibility to comprehensive adult SCD care in this region needs to be addressed promptly.^31^ Given the higher prevalence and mortality from SCD in the South region, there remains an urgent need to establish more comprehensive adult SCD centers in these areas. These centers are designed to provide multidisciplinary, patient-centered care that addresses the complex needs of patients with SCD, including preventive care such as vaccinations, disease-modifying therapies, and regular screenings for complications such as stroke and organ damage.^32,33^ Implementing these strategies can reduce acute care visits and hospitalizations, which are associated with lower mortality rates.^34,35^SCD is a genetic disorder that primarily affects African Americans in the US. In the US, African Americans often have poorer health outcomes in comparison with individuals of other races,^36,37^ which may be explained by the adverse social determinants of health among this racial group. Cha et al^38^ found that African Americans had lower rates of private insurance coverage compared with their White and Asian counterparts; this finding was exacerbated by insurance coverage disruptions during the transition from pediatric to adult care, which was associated with reduced access to preventive services and increased medication nonadherence due to cost, resulting in poorer health outcomes.^39^ Structural racism also plays a role in worse health outcomes among patients with SCD. Patients with SCD frequently encounter stigmatization and implicit bias within the health care system, leading to insufficient pain management and delays in treatment. For example, patients can be viewed as seeking drugs rather than genuinely requiring pain relief during vaso-occlusive crises.^40^ US health care policy, which structures the health care system and puts racial and ethnic minority populations at a disadvantage, also worsens the outcomes of patients with SCD. For instance, SCD receives disproportionately less research funding compared with other orphan diseases, namely cystic fibrosis. Although cystic fibrosis affects a smaller population, it receives 3.5 times more funding from the National Institutes of Health and 440 times more from other national foundations.

Our study also highlights the higher mortality rates among adult patients with SCD compared with younger age groups. This finding aligns with a study^41^ indicating a 1% rise in mortality rates among adults with SCD despite a concurrent 3% decrease in pediatric mortality rates. Another study also demonstrated that older transition-aged patients had higher ED visit and hospitalization rates than their younger counterparts.^42,43^ The challenges of transitioning from pediatric to adult care may contribute to this disparity, which may be due to the limited access to outpatient care among adults with SCD.^43^ Additionally, underutilization of hydroxyurea may further exacerbate mortality risks among adult patients. Stettler and colleagues^7^ found that more than 77% of adult patients with SCD with pain crises did not receive hydroxyurea, which is the recommended treatment. This finding is supported by another study by Treadwell et al,^11^ which demonstrated that individuals aged 26 years and older exhibited a decreased likelihood of hydroxyurea utilization compared with younger age groups. The cited barriers included patients worrying about adverse effects and patients’ forgetfulness.^11^

The SVI encompasses various social factors, including socioeconomic status, household composition, disability, minority status, and language, as well as housing type and transportation. Supported by evidence from current literature, our study demonstrates that despite advancements in medical therapy for SCD, health inequities persist. Patients from areas with higher SVI faced up to 6 times higher risk of death compared with those from areas with lower SVI. Addressing these inequities requires multifaceted and comprehensive measures, particularly in ensuring that therapy is accessible to patients across different SVI levels. These efforts are crucial to ensure that advancements in SCD intervention translate into population-level improvements in mortality rates.

### Limitations

Our study has several limitations. First, the database lacks patient-level data that include significant confounders of mortality, including the coexisting comorbidity and the treatment received. Next, SCD is relatively less common among Hispanic individuals and racial groups other than African American. CDC-WONDER will mark the data unreliable when the death count is less than 20, which may have impacted the total number of cases available for our study. However, the number was very small and less likely to reduce our ability to examine the trend analyses accurately. Our database was also limited to providing information on the location of death for our study population, without data on their domicile location. In addition, this study relied heavily on accuracy in coding due to the nature of the CDC WONDER database, which captures databased on death certificates. We also recognized that some advancements, including the approval of voxelotor and crizanlizumab, were relatively recent compared with our study period, warranting more time for evaluating their impact on sickle cell mortality at the population level. Furthermore, the lack of an option to choose the denominator for AAMR calculation, such as per 1 000 000 in the general population instead of per 1 000 000 patients with SCD, could lead to an underestimation of the absolute mortality rate of SCD. Despite the aforementioned limitations, our study sufficiently provides critical information on the association of SVI with SCD mortality. Our study methodology also has been validated in other similar research topics.^18,44^

## Conclusions

In this cross-sectional study of the association of SVI with SCD mortality rates, increased SVI was associated with increased mortality among patients with SCD. This finding highlights the ultimate need to enhance access to specialized and comprehensive SCD care because treatment advancements alone do not directly translate to improvement in nationwide mortality. Moreover, there is a critical need for the US federal government to start rendering support to establish comprehensive sickle cell centers around the country, especially in the large metropolitan areas and the South and Northeast region, where the majority of the vulnerable population of patients reside. Ultimately, comprehensive sickle cell centers will be needed nationwide to ensure equitable care and access for all affected individuals.

## Notes

Supplement 1. **eFigure 1.** 15 Social Factors Associated With the Socioeconomic Status **eFigure 2.** Different Levels of Social Vulnerability Index in Different States **eTable.** Comparison of AAMR Among Various Sex, Racial, and Geographic Subgroups

Supplement 2.
Data Sharing Statement
