# Supplementary material for: Social Vulnerability and Sickle Cell Disease Mortality in the US
Source: JAMA Netw Open. 2024 Sep 30;7(9):e2440599. doi: 10.1001/jamanetworkopen.2024.40599 (PMC11443353; doi:10.1001/jamanetworkopen.2024.40599)
Supplement: Supplement 1. — eFigure 1. 15 Social Factors Associated With the Socioeconomic Status eFigure 2. Different Levels of Social Vulnerability Index in Different States eTable. Comparison of AAMR Among Various Sex, Racial, and Geographic Subgroups [file jamanetwopen-e2440599-s001.pdf]

Supplemental Online Content

Tan JY, San BJ, Yeo Y-H, et al. Social vulnerability and isckle cell disease mortality in the US. *JAMA Netw. Open*. 2024;7(9):e2440599. doi:10.1001/jamanetworkopen.2024.40599

eFigure 1. 15 Social Factors Associated With the Socioeconomic Status

eFigure 2. Different Levels of Social Vulnerability Index in Different States

eTable. Comparison of AAMR Among Various Sex, Racial, and Geographic Subgroups

This supplemental material has been provided by the authors to give readers additional information about their work.

© 2024 Tan JY et al. *JAMA Network Open.*

eFigure 1. 15 Social Factors Associated With the Socioeconomic Status

Socioeconomic status	• Below poverty<br>• Unemployed<br>• Income<br>• No high school diploma
Household Composition & Disability	• Aged 65 or older<br>• Aged 17 or younger<br>• Civilian with a disability<br>• Single-parent households
Minority Status & Language	• Minority<br>• Speaks English “Less than Well”
Housing Type & Transportation	• Multi-Unit Structures<br>• Mobile homes<br>• Crowding<br>• No vehicle<br>• Group quarters

© 2024 Tan JY et al. *JAMA Network Open.*

eFigure 2. Different Levels of Social Vulnerability Index in Different States

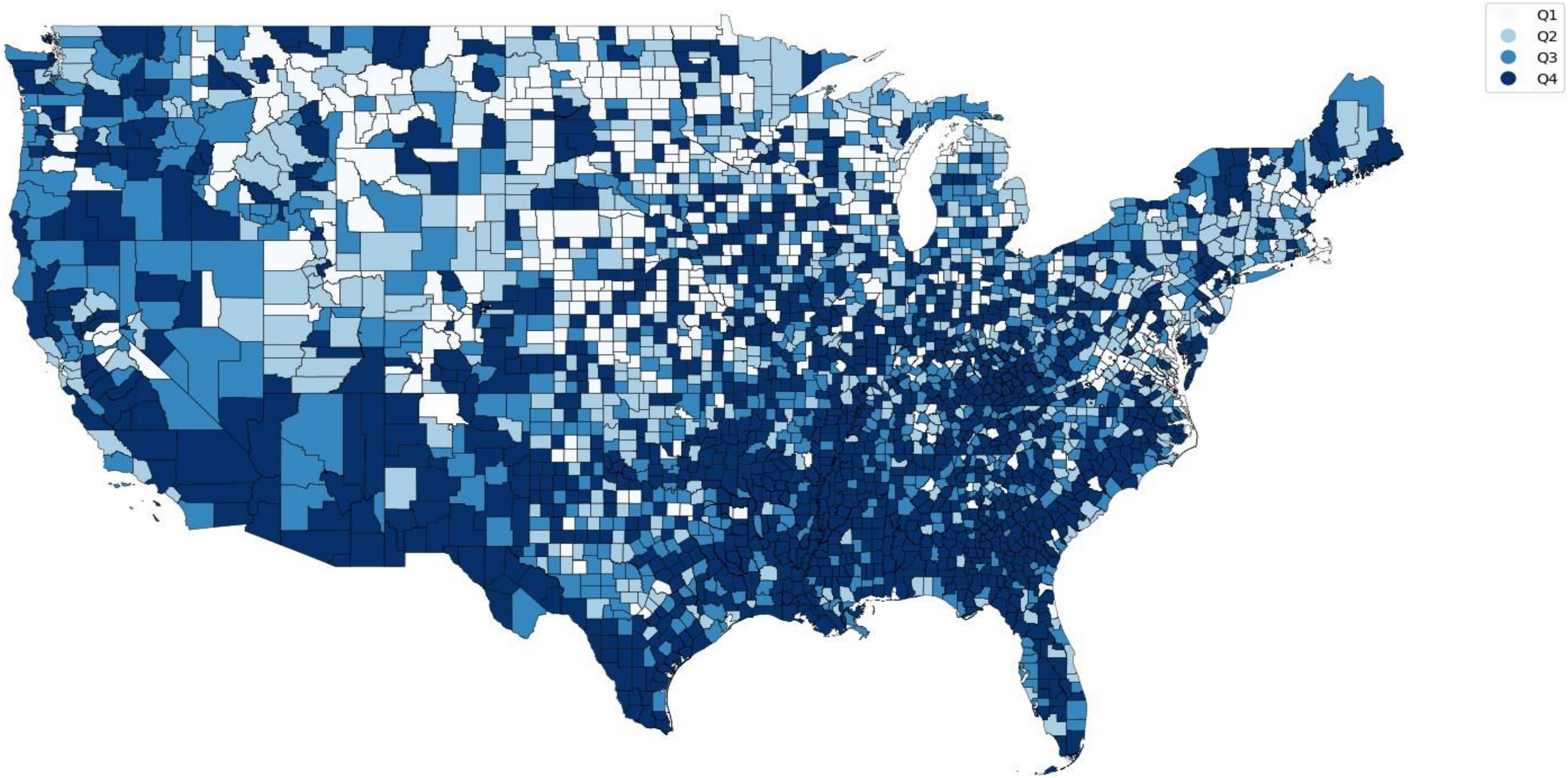

© 2024 Tan JY et al. *JAMA Network Open.*

eTable. Comparison of AAMR Among Various Sex, Racial, and Geographic Subgroups

Sickle Cell Disease (SCD) Mortality
Age-Adjusted Mortality Rates (AAMR) per 1,000,000 Individuals

Sex

Total Deaths	Male	Female
Total Deaths: 2,588	1.64 (95% CI, 1.55 - 1.73)	1.62 (95% CI, 1.53 - 1.71)

Geographic Regions - Census

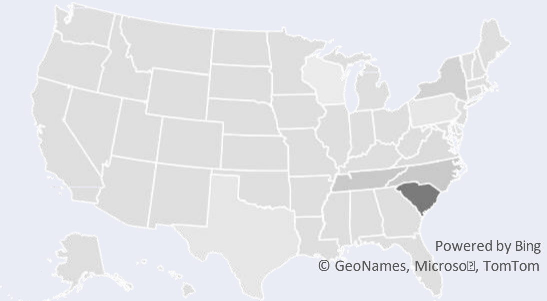

Region	AAMR
Northeast	1.58 (95% CI, 1.43 - 1.74)
Midwest	1.33 (95% CI, 1.20 - 1.45)
South	2.51 (95% CI, 2.38 - 2.64)
West	0.59 (95% CI, 0.51 - 0.67)

Geographic Regions - Urbanization Level

Urbanization Level	AAMR
Metropolitan	1.69 (95% CI, 1.62 - 1.25)
Non-Metropolitan	1.25 (95% CI, 1.10 - 1.41)

© 2024 Tan JY et al. *JAMA Network Open.*
