# Supplementary material for: Social Vulnerability and Sickle Cell Disease Mortality in the US
Source: JAMA Netw Open. 2024 Sep 30;7(9):e2440599. doi: 10.1001/jamanetworkopen.2024.40599 (PMC11443353; doi:10.1001/jamanetworkopen.2024.40599)
Supplement: Supplement 2. — Data Sharing Statement [file jamanetwopen-e2440599-s002.pdf]

# Data Sharing Statement

Tan. Social Vulnerability and Sickle Cell Disease Mortality in the US. *JAMA Netw Open*. Published September 30, 2024. doi:10.1001/jamanetworkopen.2024.40599

**Data**
**Data available:** Yes
**Data types:** Deidentified participant data
**How to access data:** https://wonder.cdc.gov/ and ATSDR, https://www.atsdr.cdc.gov/placeandhealth/svi/index.html
**When available:** With publication

**Supporting Documents**
**Document types:** None

**Additional Information**
**Who can access the data:** anyone requesting the data
**Types of analyses:** Descriptive analysis
**Mechanisms of data availability:** Publicly available
